# Beyond the Index Case: A Practical Framework for Cascade Family Screening in Hereditary Angioedema

**DOI:** 10.1007/s12016-026-09206-x

**Published:** 2026-10-01

**Authors:** Gabriel KC Leung, Andy KC Kan, Philip H Li

**Affiliations:** 1 Division of Rheumatology & Clinical Immunology, Department of Medicine, Queen Mary Hospital, University of Hong Kong, Hong Kong SAR, China; 2 Division of Rheumatology & Clinical Immunology, Department of Medicine, University of Hong Kong-Shenzhen Hospital, Shenzhen, Guangdong China

**Keywords:** Hereditary angioedema, Cascade family screening, Dried blood spot, Diagnostic algorithm, Early diagnosis

## Abstract

Hereditary angioedema (HAE) is a genetic disorder characterised by recurrent cutaneous or submucosal oedema. Most cases are caused by pathogenic *SERPING1* variants (types I and II), while a smaller subset presents with normal C1-inhibitor (C1-INH) levels and function (HAE-nC1INH). Despite its potentially life-threatening attacks, HAE remains under-diagnosed owing to inconsistent screening practices, low clinical awareness, poor patient acceptance, and resource limitations, resulting in diagnostic delays and preventable morbidity. Although international guidelines recommend family screening to identify at-risk relatives, no standardised protocol exists for its systematic implementation. Cascade family screening offers a structured approach to trace and test all known at-risk relatives across generations. Complementing this strategy, dried blood spot (DBS) assays provide a less invasive, cost-effective alternative to conventional serological testing, with particular utility in resource-limited settings. In this review, we critically appraise current screening methodologies, synthesise emerging evidence on cascade family screening and DBS-based diagnostics, and propose an actionable clinical flowchart to standardise identification, testing, and patient education from index case detection through multi-generational family screening.

## Introduction

Hereditary angioedema (HAE) is an autosomal dominant disease usually caused by a mutation in the *SERPING1* gene, resulting in deficiency or dysfunction of the C1-inhibitor (C1-INH) protein [1]. HAE can be categorised into three types based on C1-inhibitor status: type I HAE for patients with C1-inhibitor deficiency, type II HAE for patients those with C1-INH dysfunction but normal levels of C1-INH, and HAE-nC1INH for patients with normal C1-INH levels and activity (previously referred to as “type III”) caused by non-*SERPING1* genetic defects (e.g. factor XII [*F12*], plasminogen [*PLG*] and angiopoietin-1 [*ANGPT1*], kininogen 1 [*KNG1*], myoferlin [*MYOF*], heparan sulfate 3-O-sulfotransferase 6 [*HS3ST6*]), or aetiologies yet to be identified [1–3]. HAE attacks usually consist of spontaneous angioedema in the skin, upper airway and/or gastrointestinal tract, resulting in non-specific symptoms of swelling, oedema-related pain, abdominal pain, fatigue and rash amongst others; severe attacks can also lead to life-threatening conditions, such as asphyxiation in the case of laryngeal oedema blocking the airway [4]. However, a lack of awareness of HAE by patients and physicians can delay the diagnosis of patients by years and sometimes entire lifetimes, and can affect later generations of families that have carriers of the disease. HAE is also detrimental to a patient’s quality of life, where the looming risk of sudden angioedema attacks can lead to depression, stress and anxiety [5].

Globally, HAE has an overall prevalence of 0.13 to 1.6 cases per 100,000 population [5], with substantial regional difference. In resource-limited settings or regions with suboptimal awareness or access to diagnostic tests, reported prevalence can be as low as 0.02 per 100,000 [6]. Even among ethnically similar populations, estimates vary widely; in Hong Kong and the Chinese Mainland, for example, reported prevalence is 0.60 vs. 0.04 per 100,000, respectively [7, 8]. This heterogeneity likely reflects disparities in case identification and diagnostic capacity rather than true differences in disease frequency. Consequently, many patients remain undiagnosed and at risk of life-threatening complications without appropriate treatment or genetic counselling.

There is an urgent need to improve HAE screening. However, while international guidelines consistently advocate for family screening, a critical gap remains: the absence of standardised protocols detailing exactly how screening should be systematically conducted. Moreover, the costs of multiple screening tests (biochemical, immunological, and genetic) and the stringent handling requirements for traditional blood samples hinder accessibility, particularly in resource-limited or remote settings [9]. Consequently, screening practices remain highly variable and under-utilised, contributing to years-long diagnostic delays and increased mortality risk, particularly for asymptomatic relatives.

Cascade screening is a systematic approach to family screening involving screening of all at-risk relatives of the index patient, then repeating the process for each of the patients subsequently identified through screening [10, 11]. It is a reliable methodology to maximise diagnostic yield across multiple generations and a promising backbone for a standardised HAE screening protocol. Dried blood spot (DBS) assays have emerged in recent years as less invasive, highly stable, and cost-effective alternatives to conventional plasma assays. They are particularly useful in resource-constrained regions and may improve screening acceptance. Incorporating DBS assays into a standardised cascade screening protocol for HAE has been demonstrated as a novel and practical solution to these clinical practice gaps.

In this review, we evaluate current HAE screening practices and summarise the latest evidence on cascade screening and DBS assays. Furthermore, we propose an actionable clinical management flowchart that standardises the screening pathway from index case identification through multi-generational testing and patient education.

References for this review article were identified by searches of PubMed using the search terms ‘hereditary angioedema’, ‘screening’, ‘family’, ‘cascade screening’, ‘dried blood spot’, and ‘genetic’, as well as from the authors’ personal collection of literature. The final list of references was determined on the basis of relevance to the focus of this review article. The relevant summaries of the key references are illustrated in Table 1.

**Table 1:** Key references and their relevant summary

Title	Author	Year	Relevant Summary
Diagnosis and screening of patients with hereditary angioedema in primary care [12]	Henao et al.	2016	- Family screening of first-degree family members is encouraged
Complement factor C4 activation in patients with hereditary angioedema [13]	Aabom et al.	2017	- Investigates the use of C4c/C4 ratio in diagnosis compared to total antigenic C4
Mutational spectrum of the SERPING1 gene in Swiss patients with hereditary angioedema [14]	Steiner et al.	2017	- Investigates the mutational spectrum of HAE in unrelated Swiss families
The physician and hereditary angioedema friend or foe: 62-year diagnostic delay and iatrogenic procedures [15]	Valeviera et al.	2018	- Demonstrates lack of awareness of HAE even with family history, only diagnosed after patient’s initiative into researching symptoms - Subsequent family screening revealed undiagnosed HAE spanning three generations
A hereditary angioedema screening in two villages, based on an index case, and identification of a novel mutation, “1033G > T”, at the SERPING1 gene [16]	Ozkars et al.	2019	- Familial screening used to identify cases of HAE after family pedigree construction, with a majority being paediatric patients
A novel functional C1 inhibitor activity assay in dried blood spot for diagnosis of Hereditary angioedema [17]	Lai et al.	2020	- Development and validation of a functional C1-INH assay using dried blood spots
Screening for Plasminogen Mutations in Hereditary Angioedema Patients [18]	Farkas et al.	2021	- Case report using first-degree family screening for PLG-HAE
Diagnosing Pediatric Patients With Hereditary C1-Inhibitor Deficiency—Experience From the Hungarian Angioedema Center of Reference and Excellence [19]	Andrási et al.	2022	- Retrospective analysis of paediatric HAE diagnosis, of which a majority was done via family screening
The international WAO/EAACI guideline for the management of hereditary angioedema-The 2021 revision and update [1]	Maurer et al.	2022	- Use of C4 and C1-INH testing supported - Family screening is recommended, with no further elaboration on methodology
A Missense Mutation of the Plasminogen Gene in a Japanese Family with Hereditary Angioedema with Normal C1 Inhibitor: Third Family Survey in Asia [20]	Yakushiji et al.	2023	- Case report using first-degree family screening for PLG-HAE
Prospective Study on the Efficacy and Impact of Cascade Screening and Evaluation of Hereditary Angioedema (CaSE-HAE) [8]	Wong et al.	2022	- Employment of cascade family screening to screen family members of HAE patients, with approximately 50% newly diagnosed
Application of a dried blood spot based proteomic and genetic assay for diagnosing hereditary angioedema [21]	Iuraşcu et al.	2023	- Validation of dried blood spot assays for C4, C1-INH and genetic analysis in HAE diagnosis
Screening for type II hereditary angioedema—the “poor man’s c1-inhibitor function” [22]	Jindal et al.	2023	- Variation in C1-INH levels between family members with the same mutation
National survey on clinical and genetic characteristics of patients with hereditary angioedema in Latvia [23]	Kanepa et al.	2023	- Highlights importance of established family screening procedure and assay availability
Uncovering the true burden of hereditary angioedema due to C1-inhibitor deficiency: A focus on the Asia-Pacific region [6]	Honda et al.	2024	- Emphasises the benefits of family screening in disease prevalence analysis - Explores reasons for late diagnosis despite family history and related consequences
Hereditary angioedema in children: Review and practical perspective for clinical management [24]	Pagnier et al.	2024	- Review of HAE screening for infants, including those with HAE family history - Family history helps with early diagnosis
Validating and utilizing dried blood spots for family screening: Screening Programme Providing Outreach for Testing Hereditary Angioedema (SPPOT-HAE) [25]	Wong et al.	2025	- Use of dried blood spot assays as a follow-up to previous cascade family screening programmes, with comparison to traditional C4 assays
Early diagnosis of hereditary angioedema in children: genetic testing should be prioritized [26]	Bocquet et al.	2025	- Case report where C1-INH assays were normal in infancy, only to show reduced activity later in life, with type II HAE confirmed by genetic screening
Implementation of genetic diagnosis and personalized management of hereditary angioedema in a Chinese regional center: a community case study of three families [27]	Du et al.	2025	- Use of cascade family screening in three unrelated affected families alongside genetic counselling to create personalized treatment regimes
Cascade Screening of Hereditary Angioedema in Pakistan [28]	Hussain et al.	2025	- Employment of cascade family screening for relatives of newly diagnosed HAE patients, with 45% confirmed and probable cases identified
A nationwide questionnaire-based surveillance on pediatric hereditary angioedema in Japan [29]	Yamamoto et al.	2025	- Survey on paediatric cases of HAE, revealing that paediatric relatives of HAE patients did not undergo family screening due to hesitancy
Type II hereditary angioedema with an apparently de novo SERPING1 mutation in China: A case report and family screening [30]	Guan et al.	2026	- Case report involving the use of dried blood spot in cascade family screening from an index patient

C1-INH, C1-inhibitor; HAE, hereditary angioedema; PLG, plasminogen

### Current Screening Approaches and their Limitations

#### Conventional Biochemical and Genetic Testing

Conventionally, patients with clinically suspected HAE are tested for plasma C4 levels during and between attacks, with further assays of C1-INH antigen and function to distinguish type I from type II disease [31]. This biochemical panel is highly accurate for type I and II HAE, yet not all centres can perform all three tests, which reduces diagnostic sensitivity in resource-limited settings [1]. Type I HAE, the most common form, is characterised by low C4 and low antigenic C1-INH. However, a normal level of C1-INH cannot exclude the presence of other types of HAE. Type II HAE is identified by normal or low-normal antigenic C1-INH with reduced function. The 2025 World Allergy Organization (WAO) guidelines suggests that C1-INH functional assays alone are sufficient for diagnosing HAE-C1-INH, with C1-INH antigen assays and plasma C4 levels serving as alternative tests when C1-INH function assays are unavailable [32]. Alternatively, low C4 and normal/elevated C1-INH levels (termed the “poor man’s C1-INH function”) may be considered as a screening tool for type II HAE, especially in countries where C1-INH function testing is not readily available [22].

C4 is widely accepted as the initial screening test, yet approximately 10% of HAE patients present with normal C4 levels [12]. Aabom et al. showed that danazol and other androgen derivatives can normalise C4, masking underlying disease [13]. C4 levels also vary between individuals and fluctuate around reference values. In paediatric populations, immature complement system in newborns can yield false-positive results if screened before maturation [24]. Moreover, the sensitivity of C4 testing appears to vary by ethnicity: reported sensitivity is lower in Asian cohorts (58%) than in Western cohorts (81–96%) [8]. To address these limitations, the ratio of the activation product C4c to total antigenic C4 (C4c/C4) has been proposed as an alternative marker. Because C4c is generated during complement activation, an elevated C4c/C4 ratio indicates C4 consumption and is independent of absolute C4 concentration [13]. This makes the ratio particularly attractive for early paediatric diagnosis, as it remains interpretable even when total C4 is low because of physiological immaturity. However, its utility depends on demonstrable complement activation; it is not informative in the rare scenario where C4 is low without consumption.

For patients with normal or equivocal biochemical results, or to differentiate HAE from acquired angioedema, genetic testing is indicated. Sanger sequencing has traditionally been used to analyse *SERPING1*, or other genes that may cause HAE-nC1INH, which include factor XII (*F12*), plasminogen (*PLG*) and angiopoietin-1 (*ANGPT1*), kininogen 1 (*KNG1*), myoferlin (*MYOF*) and heparan sulfate 3-O-sulfotransferase 6 (*HS3ST6*) [1, 33]. More recently, targeted next-generation sequencing (NGS) panels, whole-exome sequencing, and whole-genome sequencing have identified novel pathogenic variants in cases without a prior genetic diagnosis [34–36].

#### Practical Barriers to Conventional Testing

These testing modalities are limited by accessibility, cost, and patient burden. Firstly, the use of a series of multiple tests from the initial C4 and C1-INH levels, followed by C1-INH function, and in equivocal cases or suspected HAE-nC1INH, genetic tests, is considerably costly. In regions lacking local laboratory capacity, samples must be shipped to external reference centres, incurring further costs and diagnostic delays [37]. Secondly, sample storage has stringent requirements. C1-INH protein is labile in vitro; unless testing is performed within hours, samples require on-site centrifugation and storage at − 20 °C, necessitating an expensive cold chain for transport [38, 39]. The interval between sample collection and plasma separation can also affect the sensitivity of the assay [40]. Thirdly, in terms of acceptance, venipuncture required for blood collection is relatively invasive and uncomfortable, which is especially relevant in the context of screening for asymptomatic individuals and if repeated blood collection is needed [41, 42].

#### Family Screening

Testing only after a patient has already experienced an attack that raises suspicion of HAE results in significant under-diagnosis of the disease. Thus, family screening has been employed to diagnose additional cases within the families of patients with HAE.

Relying solely on attack-triggered diagnosis results in substantial underdiagnosis. Family screening is therefore recommended to identify at-risk relatives of confirmed HAE patients. International guidelines advise screening first-degree relatives (parents, siblings, and children), with extension to additional relatives where appropriate [1]. Because HAE is autosomal dominant, each offspring of an affected parent has a 50% risk of inheriting the disorder. As a result, family screening is vital in identifying asymptomatic patients before their first HAE attack, as it can be fatal due to risks of asphyxiation from airway attacks. Apart from enabling prompt treatment with prophylactic and on-demand medications, it can also facilitate early family planning [43, 44].

Family screening has proven diagnostic yield. Ozkars et al. used family screening to identify 35 additional cases of undiagnosed HAE, with a majority of those cases being asymptomatic and for patients under the age of 18 [16]. This was done through family pedigree tree construction across three generations and separated in two villages. This demonstrates how family screening helps in early diagnosis, allowing for newly diagnosed patients to be educated and prepared in case of future attacks, reducing the risk of morbidity and mortality.

Although their symptoms may vary in terms of age of onset and severity, family members also tend to share the same mutation for HAE. In a study by Steiner et al. examining eight families with HAE, each family was found with a unique variant in the *SERPING1* gene that was exclusive to their family [14]. This phenomenon was also found in various other studies conducting family screening for HAE patients [16]. From this, it is possible to use the specific genotype of an index HAE patient to perform genetic family screening more cost-efficiently. This is especially the case for HAE-nC1INH, where the mutation can be located on various genes that can complicate the screening procedure; as long as the mutation can be isolated in the index patient, the same mutation can be screened for in all other family members, streamlining the process. It may also be possible to predict certain factors of HAE manifestation once the family mutation is known; mutations involving nucleotide duplication or deletion are observed to be associated with more severe clinical courses compared to missense mutations [14]. Thus, knowing the mutation type for asymptomatic family members of confirmed HAE cases can help guide treatment procedures to mediate more aggressive forms of HAE, such as increasing prophylactic measures provided.

Family screening is especially useful in the field of paediatrics. Symptom onset can occur in infancy, when children are vulnerable and unable to self-manage attacks. In practice, most paediatric cases of HAE are diagnosed by family screening stemming from an index patient relative, prior to their first HAE attack [16, 19]. *De novo* mutations, which lack a family history, remain difficult to detect pre-symptomatically without universal screening.

Biochemical screening in infants is complicated by physiological complement immaturity. C4 and C1-INH levels may not reach adult ranges until 6–36 months of age; consequently, initial testing is generally deferred to 6 months, with follow-up at 12 months [24]. A case has also been documented where infantile patients initially presenting with normal C1-INH levels had a drop in C1-INH levels following their first year of life, only detected by assessments up to 4 years later [26]. This demonstrates that screening C4 and C1-INH levels may not be sufficient in the detection of HAE in infancy. Genetic testing avoids these ambiguities and is increasingly preferred for neonatal diagnosis, as reported by Andrási and Bocquet et al. [19, 26]. This is also supported by the French organisation CRÉAK, which additionally recommended the use of buccal swabs instead of blood tests to conduct genetic screening for newborns in order to minimise neonatal distress [26].

Non-*SERPING1* variants also warrant attention in familial screening. An example is HAE caused by a mutation on the plasminogen gene (PLG-HAE), of which family cases have been detected in case reports from Japan and Hungary [18, 20]. In both cases, patients and family members demonstrated normal C4 and C1-INH activity and levels, and PLG-HAE was diagnosed only after strong suspicions of HAE-nC1INH resulted in follow-up genetic screening for various HAE-causing variants, prompting family screening for the same mutation in their respective families. Population-specific differences exist: *F12* variants account for roughly 25% of HAE-nC1INH in Caucasian populations but have not been reported in Asian cohorts, suggesting ethnic heterogeneity in HAE-nC1INH genetics [20]. As such, it is important for clinicians to be aware of relevant variants of HAE to reduce misdiagnosis.

While the evidence and appropriate tools for family screening are in place, there has been a lack of guidance on how to systematically carry out family screening. Although international guidelines recommend family screening, the absence of standardised protocols has led to highly variable practice worldwide [1, 32, 45, 46]. Some centres screen only first-degree relatives, yielding approximately 30% of at-risk cases, while others use ad hoc methods that may miss relatives across generations [47]. A systematic, protocol-based approach is needed to maximise diagnostic yield, reduce delays, and ensure equitable access to HAE screening.

### Cascade Family Screening

**Fig. 1:**
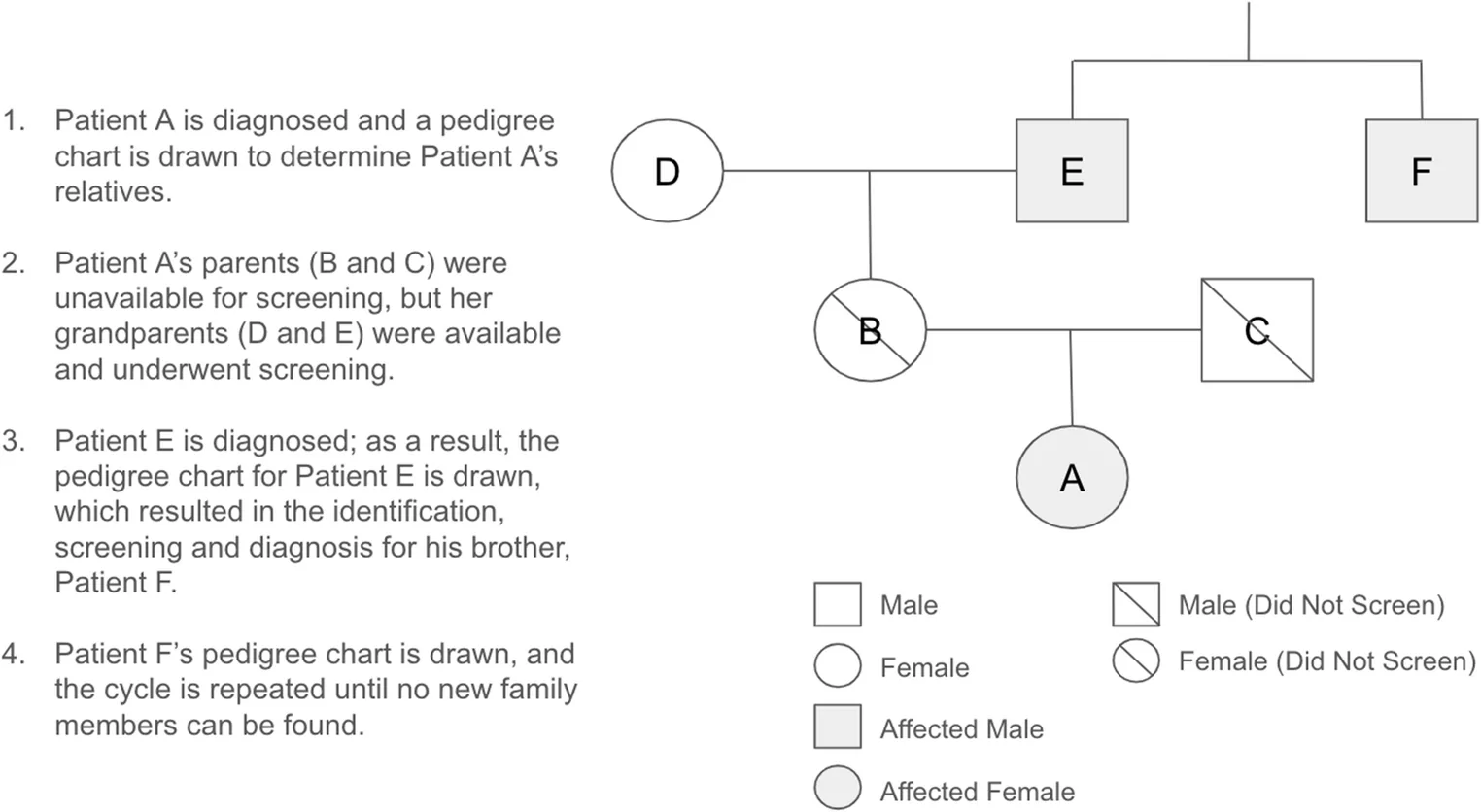
Example of cascade screening

Cascade screening is a systematic approach to family screening. Beginning with the index patient, it involves identifying all at-risk relatives of a patient for screening, the process of which is typically done through family pedigree charting [8]. The first-degree relatives of a confirmed patient are prioritised for screening, but when a potential at-risk relative is unable to or decline screening, the relatives of that individual could still be offered screening. As more patients in the extended family are diagnosed, this process repeats with more at-risk relatives identified for screening, until no other known family members can be found. An example of cascade screening is illustrated in Fig. 1. Such screening strategies have also been performed for other rare inheritable disorders such as Fabry disease, with marked success [48].

A study conducted in Hong Kong was first to demonstrate that such approach prevents at-risk individuals from being overlooked by offering testing to the descendants of relatives who decline or are unavailable; in doing so, they diagnosed 10 additional patients who would have been missed by screening restricted to first-degree relatives alone [8]. In Hong Kong, systematic case identification through the Cascade Screening and Evaluation of Hereditary Angioedema (CaSE-HAE) programme has substantially increased the number of confirmed diagnoses, bringing the ascertained prevalence closer to global figures. This suggests that earlier estimates were artificially low because of under-ascertainment rather than reflecting true epidemiological differences. Thus, cascade family screening improves not only individual patient outcomes through earlier diagnosis, but also the accuracy of regional disease surveillance.

These findings have been replicated in diverse healthcare settings. Multiple other case reports also employed cascade screening in multiple families to identify HAE patients, including studies done by Hussain et al. in Pakistan [28], and *Du et al.*. and Guan et al. in China [27, 30]. All of them identified multiple new HAE patients within the index patient’s immediate and extended family. Early detection enabled prompt treatment and genetic counselling, thereby improving quality of life and reducing mortality risk. Du et al. further showed that once a familial pathogenic variant was identified, targeted genetic screening of relatives avoided unnecessary sequencing and reduced both cost and turnaround time [27]. Together, these studies demonstrate that cascade family screening is reproducible across diverse environments and delivers both clinical and economic benefits.

### Dried Blood Spot Assays

The use of DBS testing in HAE screening has been documented by multiple studies. Whereas conventional screening requires venepuncture, DBS collection requires only a finger prick to obtain a small volume of blood spotted onto a collection card. Compared to traditional liquid samples, DBS samples have less invasive collection procedures and a significantly greater stability of 134 days at room temperature and 3 days at 45℃ for transport and storage [17]. In addition, DBS quantification of C4 and C1-INH showed the same patterns as plasma samples [21], further validated by other studies where a strong correlation was found with no false negative results [25]. These data indicate that DBS-based assays offer equivalent diagnostic performance to traditional serum assays, making them an attractive option for HAE screening.

DBS testing can also be used for genetic screening. Iuraşcu et al. used standard spin-column-based extraction methods to isolate DNA from DBS samples and successfully screened for known pathogenic variants in the *SERPING1* gene [21]. This allows simultaneous biochemical screening and genetic confirmation from a single DBS sample, potentially minimising diagnostic delays arising from the transport and turnaround times of multiple conventional blood tests. This is particularly valuable in remote areas or regions lacking tertiary immunology services.

Due to its less invasive nature, DBS can be a more appealing option for patients who are unable or unwilling to undergo a traditional blood test, especially for family screening processes where identification of HAE patients is of key importance. Wong et al. demonstrated this phenomenon in their study, where multiple members of a family with HAE patients who once persistently declined to undergo genetic screening in a past study were able to be persuaded to undergo DBS screening instead, of which two relatives were found to have low C4 and low C1-INH levels [25].

DBS testing is not limited to type I HAE. Guan et al. employed DBS within a cascade family screening programme for type II HAE and identified three affected relatives, two of whom were asymptomatic [30]. Published data on DBS testing in HAE-nC1INH are currently lacking; however, given the established feasibility of DBS-based genetic analysis, it is reasonable to anticipate that the platform would be applicable to screening for non-*SERPING1* variants as well.

In summary, DBS assay represents a promising platform in which all testing could potentially be done with a single DBS sample, replacing multiple blood samples for various tests. Its stability, without the need for a cold chain during transport, could minimise costs and the risk of sample damage, which are especially relevant for resource-limited and remote countries or regions. The minimally invasive collection procedure may also improve screening acceptance and uptake.

### A Proposed Structured Clinical Management Flowchart for HAE Family Screening

**Fig. 2:**
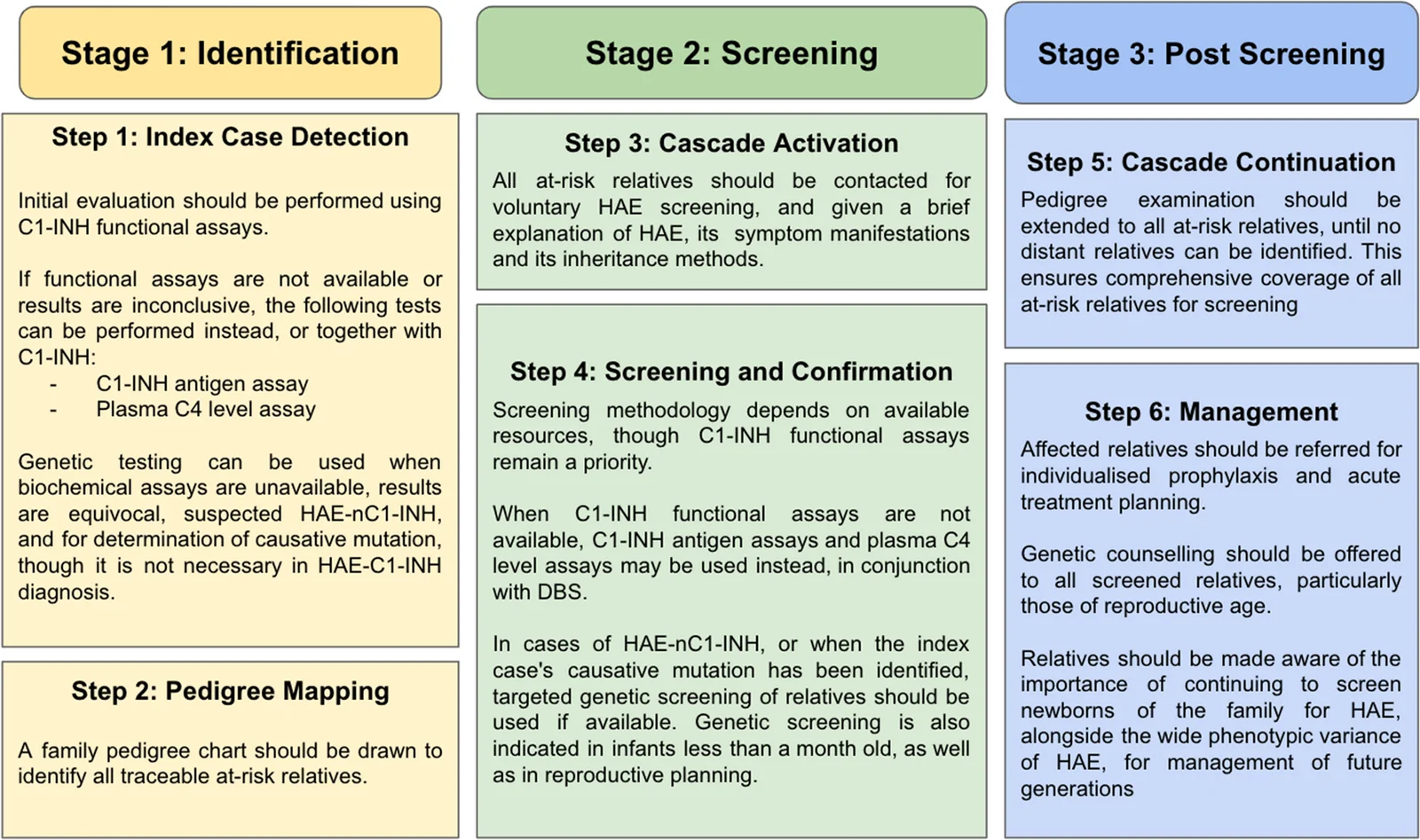
Proposed clinical management flowchart for cascade screening of HAE

Despite international guidelines advocating family screening in HAE, none has outlined a specific procedure for its systematic conduct [1, 32, 45, 46]. This absence of a standardised protocol likely contributes to the limited global adoption of family screening, as providers must conduct screening based on their own research and knowledge of HAE. Although sample collection through venepuncture or DBS sampling is straightforward and can be readily integrated into routine clinical practice, not every region has immediate access to C1-INH and C4 assays or genetic testing; establishing the necessary infrastructure for such tests will require time. Drawing on the evidence reviewed above, alongside the most recent WAO guidelines, we have devised a structured clinical management flowchart for HAE family screening (Fig. 2) [32].

#### Step 1: Index Case Detection

Because HAE attacks are episodic and patients may be asymptomatic between episodes, diagnosis is usually triggered by a symptomatic presentation. In terms of priority, initial evaluation should prioritise C1-INH functional assays, followed in order by C1-INH antigens and plasma C4 level, depending on regional availability and necessity. Genetic testing may be used as a first-line approach where biochemical assays are unavailable or where results are equivocal, especially for diagnosis of suspected HAE-nC1INH, though it is not required for HAE-C1-INH diagnosis. Genetic testing may also be used as a follow-up to HAE diagnosis for identification of the causative mutation, which can aid future cascade screening. The differences in methodology between index-case evaluation and family screening are summarised in Table 2.

**Table 2:** Differences between index and family screening

	Index Screening	Family Screening
**Purpose**	Identify possible HAE patients with compatible symptoms	Identify further HAE patients among at-risk relatives of known patients
**Trigger**	Symptomatic attack and clinical suspicion	Confirmed diagnosis in index case
**Clinical context**	Patient often symptomatic at time of screening	Can be symptomatic (undiagnosed) or asymptomatic at time of screening
**Biochemical testing**	C4 level ± C1-INH level ± function	C1-INH level ± function (preferred over C4 alone)
**Genetic testing**	HAE Gene panels, often reserved for equivocal tests or HAE-nC1INH	Targeted screening (Sanger) for known familial mutation

#### Step 2: Pedigree Mapping and Relative Tracing

Once an index case is confirmed, a family pedigree chart should be constructed to identify all traceable at-risk relatives, as demonstrated by Ozkar and Wong et al. [8, 16].

#### Step 3: Cascade Activation and Education

All at-risk relatives should be contacted for voluntary HAE screening, during which a brief explanation of HAE, its symptom manifestations and its inheritance methods should be provided to promote awareness of HAE.

#### Step 4: Screening and Confirming

Screening methods can vary depending on available resources; for HAE-C1-INH, conventional C1-INH assays remain reliable, though alternate options such as DBS assays may also be used in areas where such assays cannot be immediately analysed and require longer-term storage and transport to facilities with the necessary resources. C4 screening is less advisable as other methods are more specific to known HAE types, though still feasible as an alternative in areas where C1-INH is not available. In cases of HAE-nC1INH, or when the index case’s causative mutation has been identified, targeted genetic screening of relatives is more specific than biochemical testing alone and can be performed using either conventional blood samples or DBS. For infant relatives, biochemical testing is unreliable before 6–12 months of age; genetic screening is therefore preferred, with biochemical confirmation deferred until 12 months of age if genetic testing is unavailable.

#### Step 5: Cascade Continuation

We recommend cascade family screening, extending pedigree analysis to all traceable at-risk relatives. This should be done by drawing pedigree charts of all the index patient’s relatives and continuing until no other distant relatives of the index patient can be identified.

#### Step 6: Post-Screening Management

Affected relatives should be referred for individualised prophylaxis and acute treatment planning. Genetic counselling should be offered to all screened relatives, particularly those of reproductive age. Family members should be counselled on the importance of ongoing newborn screening for HAE. Given the condition’s wide phenotypic variability, this continuous screening is essential for effective disease management in future generations.

### Organizational Framework for Cascade Screening

The effectiveness of cascade screening is often limited by a lack of coordination, limited awareness of HAE, and inequitable access to specialized diagnostic testing. To overcome these challenges and facilitate screening for geographically dispersed relatives, we propose a ‘Hub-and-Spoke’ organizational framework.

At the core of this model, national “Hubs” consisting of HAE centres of excellences (e.g. Angioedema Centres of Reference and Excellence [ACARE]) would maintain centralized registries of HAE families and oversee nationwide screening efforts. These national Hubs act as referral centres for advanced diagnostic methodologies that would be more difficult to perform in remote locations. Operating at the next tier would be regional hubs (such as tertiary referral centres with HAE experience and clinical immunology expertise). These regional centres bridge the gap by assisting national hubs in reporting new cases, coordinating regional family screening, and providing more accessible specialist care. The “Spokes” would consist of local, non-specialist clinics that have completed accredited HAE training from their Hubs. Serving as accessible primary points of contact, these spokes can also evaluate suspected patients and reach out to at-risk relatives. For example, patients can have their samples collected locally using DBS, which are then transported to their hubs for diagnostic testing. If these initial results are positive, patients can then be referred to regional hubs for confirmatory testing. This tiered system would help reduce the unnecessary logistical burden, eliminating the need for at-risk relatives to travel long distances to specialized centres for initial screening.

Furthermore, this model establishes a vital knowledge exchange network. Hubs can provide continuous medical education for trained non-specialist physicians at the spokes, empowering these local practitioners to effectively identify and triage suspected cases of HAE. Simultaneously, the spokes facilitate patient education and empowerment. Ultimately, this bidirectional exchange improves regional diagnostics, fosters local research, and equips patients with the knowledge needed for effective self-management and improved quality of life.

### Knowledge Gaps and Future Research Directions

Literature on screening for HAE-nC1INH variants remains sparse. In the studies we reviewed, only Yakushiji and Farkas et al. reported family screening in PLG-HAE [18, 20]. Although HAE-nC1INH accounts for a small fraction of all HAE cases, these studies demonstrate that PLG variants can be inherited, underscoring the importance of family genetic screening for this subtype. Incorporating HAE-nC1INH into cascade family screening protocols would not only improve case detection and patient outcomes, but would also generate valuable case series to advance understanding of the pathophysiology and phenotypic spectrum of these rare variants. DBS assays may streamline this process. Although, to our knowledge, no published study has yet used DBS for genetic screening of non-*SERPING1* HAE, it has been shown that DBS samples can reliably be used for targeted mutation analysis [21]. Applying this platform to HAE-nC1INH screening is a logical next step.

Psychosocial and socioeconomic outcomes of screening remain understudied. In the literature we reviewed, only one study examined the mental and financial burdens of HAE in depth. Few studies have assessed how quality of life changes after relatives undergo screening. At-risk family members often experience significant anxiety about inheriting the disorder, which itself impairs quality of life. Wong et al. found that although depression scores did not change after screening, anxiety improved significantly in both symptomatic and asymptomatic screened relatives [8]. This was attributed to patient empowerment: a definitive diagnosis provides disease understanding, treatment access, and improved self-management. Wong et al. also reported that cascade family screening was cost-effective, saving up over USD $150 per person annually through a 70% reduction in HAE-related hospitalisation costs driven by early diagnosis and better symptom control. Because DBS sampling is less invasive and more stable than conventional venepuncture, future studies should incorporate DBS into cascade protocols to further improve cost-effectiveness and reduce the psychosocial burden of screening.

The most frequently cited barrier to HAE screening is poor disease awareness among both the general public and physicians, compounded by patient reluctance to undergo testing. These factors contribute to diagnostic delays of months to years. Patients experiencing acute attacks frequently present to emergency departments, where HAE is often misdiagnosed because emergency physicians fail to recognise the condition [49]. In some regions, routine family screening is not offered to confirmed HAE patients [15, 23], likely because diagnosing physicians are unfamiliar with the inheritance pattern. Honda et al. examined this issue in depth in the Asia-Pacific region, identifying multiple contributing factors: a shortage of trained immunologists, limited testing capacity, and inadequate national health insurance coverage [6]. All of these barriers to screening can delay diagnosis, placing patients at greater risk of mortality from the unpredictable nature of HAE attacks.

Patient hesitancy is also a major issue in family screening; Wong and Yamamoto et al. both reported that several at-risk relatives declined screening [8, 29]. Reasons are likely multifactorial and may include poor understanding of HAE, anxiety about results, time constraints, discomfort with venepuncture, or fear of genetic discrimination [49]. Hence, it is vital that awareness of HAE and its symptoms be raised through both the general public and the medical community, both to destigmatise diseases such as HAE that can alter a patient’s appearance and to promote the importance of screening and the early diagnosis of HAE. Better public understanding would enable symptomatic individuals to seek appropriate testing sooner, while improved physician education would reduce diagnostic delay and ensure timely treatment and support.

The success of pilot studies employing cascade screening and DBS assays paves the way for large-scale, multi-centre, international trials using a standardised protocol. High-quality evidence is needed to support incorporation of such protocols into international guidelines. Future research should also evaluate broader implementation outcomes, including cost-effectiveness and quality-of-life impacts for patients and their families.

## Conclusion

In conclusion, enhancing the diagnosis and management of HAE warrants a shift from reactive, symptom-driven testing to proactive, systematic family screening guided by standardised protocols. Cascade screening, coupled with innovative, less invasive methods such as DBS assays, holds significant promise in identifying asymptomatic patients early, thus reducing diagnostic delays, preventing life-threatening attacks, and improving quality of life. Implementing a structured clinical flowchart for family screening could facilitate consistency, efficiency, and scalability across diverse healthcare settings. To realise the full potential of these strategies, concerted efforts are required to raise awareness among healthcare providers and the public, address existing knowledge gaps, particularly concerning HAE variants beyond *SERPING1*, and investigate the cost-effectiveness and psychosocial benefits of standardised screening protocols. Ultimately, embedding these approaches into clinical practice and guidelines will be pivotal in improving patient outcomes and family well-being across diverse healthcare settings.

## Data Availability

No datasets were generated or analysed during the current study.
